# Phenolic Compounds Recovery from Blood Orange Peels Using a Novel Green Infrared Technology *Ired-Irrad*^®^, and Their Effect on the Inhibition of *Aspergillus flavus* Proliferation and Aflatoxin B1 Production

**DOI:** 10.3390/molecules27228061

**Published:** 2022-11-20

**Authors:** Sally El Kantar, Hiba N. Rajha, André El Khoury, Mohamed Koubaa, Simon Nachef, Espérance Debs, Richard G. Maroun, Nicolas Louka

**Affiliations:** 1 Université de Technologie de Compiègne, ESCOM, TIMR (Integrated Transformations of Renewable Matter), Centre de Recherche Royallieu, CS 60319, 60203 Compiègne Cedex, France; 2 Centre d’Analyses et de Recherche, Unité de Recherche Technologies et Valorisation Agro-Alimentaire, Faculté des Sciences, Université Saint-Joseph de Beyrouth, Riad El Solh, P.O. Box 17-5208, Beirut 1104 2020, Lebanon; 3 Ecole Supérieure d’Ingénieurs de Beyrouth (ESIB), Université Saint-Joseph de Beyrouth, CST Mkalles Mar Roukos, Riad El Solh, P.O. Box 11-514, Beirut 1107 2050, Lebanon; 4 Techno Heat Society, Al Firdaws Street, Sabtiyeh, Beirut 1100, Lebanon; 5 Department of Biology, Faculty of Arts and Sciences, University of Balamand, P.O. Box 100, Tripoli 1300, Lebanon

**Keywords:** blood orange peels, phenolic compounds, infrared-assisted extraction, optimization, Aflatoxin B1

## Abstract

The intensification of total phenolic compound (TPC) extraction from blood orange peels was optimized using a novel green infrared-assisted extraction technique (IRAE, *Ired-Irrad^®^*) and compared to the conventional extraction using a water bath (WB). Response surface methodology (RSM) allowed for the optimization of ethanol concentration (E), time (t), and temperature (T) in terms of extracted TPC and their antiradical activity, for both WB extraction and IRAE. Using WB extraction, the multiple response optimums as obtained after 4 h at 73 °C and using 79% ethanol/water were 1.67 g GAE/100 g for TPC and 59% as DPPH inhibition percentage. IRAE increased the extraction of TPC by 18% using 52% ethanol/water after less than 1 h at 79 °C. This novel technology has the advantage of being easily scalable for industrial usage. HPLC analysis showed that IRAE enhanced the recovery of gallic acid, resveratrol, quercetin, caffeic acid, and hesperidin. IR extracts exhibited high bioactivity by inhibiting the production of Aflatoxin B1 by 98.9%.

## 1. Introduction

Phenolic compounds are plant secondary metabolites, known for their antioxidant, antimicrobial, anticarcinogenic and anti-inflammatory activities [1,2]. In food industries, they prevent the rancidity of oils and fats [3], and can inhibit fungal growth and subsequent mycotoxin production in stored grains [4].

Aflatoxins are toxic secondary metabolites especially produced by two filamentous fungi *Aspergillus flavus* and *Aspergillus parasiticus* [5,6]. Under favorable conditions of humidity and temperature, these fungi grow on foods and feeds like corn, peanuts, cottonseed and cereals, resulting in aflatoxins production [7]. Aflatoxin B1 (AFB1), the most potent and toxic one, is a mutagenic, carcinogenic, teratogenic and immunodepressive agent. Humans and animals are exposed to AFB1 through ingestion, skin contact and inhalation [8]. Pesticides are usually used to protect crops from the development of fungi. However synthetic fungicide residues also have toxic effects, and pathogens can develop resistance to it. For these reasons, interest in the use of natural antimicrobials such as essential oils and phenolic compounds is growing [9].

*Citrus* (*Rutaceae* family) is an important world fruit crop [10]. Its health benefits are mainly related to the presence of bioactive compounds, such as polyphenols, vitamin C and carotenoids [11,12]. After consuming citrus fruits as fresh produce or juice, a large number of peels are generated and generally discarded as waste [13], used as animal feed or for fuel production [14]. The extraction of total phenolic compounds (TPC) from citrus peels is attracting more and more attention due to their biological virtues as natural antioxidants and antimicrobials [15]. Conventional methods such as water bath (WB) extraction, were used for the valorization of citrus peels by the extraction of bioactive compounds [16]. Nonetheless, researchers have been focusing on developing innovative extraction techniques for industries that can be more efficient and energy-saving [17,18]. Extraction techniques such as ultrasound [19], microwave [20,21], instantaneous controlled pressure drop [22,23], supercritical CO_2_ [24], pulsed electric field [25,26] and Intensification of Vaporization by Decompression to the Vacuum (IVDV) [27] were previously used to intensify the extraction of bioactive compounds from agro-industrial residues and by-products. These extraction processes can improve mass transfer, decrease the extraction time and temperature and reduce solvent use. They can also improve the recovery of bioactive compounds from by-products with lower energy consumption.

The green extraction process is based on the discovery and the design of techniques to reduce energy and solvent consumption [28]. In this sense, infrared-assisted extraction (IRAE) technology is a novel, simple and low-cost extraction method that can be scalable to industrial level. IR radiation is characterized by its high penetration ability and has found many applications in health care and industrial treatment [29].

To the best of our knowledge, IR was not previously tested for the extraction of polyphenols from blood orange peels. The objective of this work is to test the efficiency of this novel green technology for the recovery of high TPC yields from blood orange peels and to investigate the potential use of the obtained extracts as antifungal and anti-mycotoxigenic agents. The optimization of TPC extraction was conducted by a conventional WB extraction and a novel method of IRAE. The optimal ethanol concentration, time, and temperature were determined by response surface methodology (RSM) for the two techniques. Chemical characterization of the extracted TPC was also explored.

## 2. Results and Discussion

Time (t), temperature (T) and ethanol percentage (E) were optimized for WB and IR extraction of TPC from blood orange peels using RSM. The latter is an effective statistical optimization method. It allows the evaluation of multiple parameters and their interactions with a reduced number of experiments. Table 1 and Table 2 show the experimental design, where factors are presented in their real and coded values, with their responses TPC and inhibition % of DPPH, for the conventional WB extraction and IRAE. For WB extraction, TPC ranged from 1.20 to 1.67 g GAE/100 g DM, and inhibition percentage from 37.6% to 58.7%. While for IRAE, TPC ranged from 0.95 to 1.90 g GAE/100 g DM (14% increase compared to WB) and inhibition percentage from 34.7% to 56.4%. The obtained results are comparable to those obtained in a previous study where the yields of polyphenols extracted from orange peels ranged between 0.30 and 1.70 g GAE/100 g DM with DPPH inhibition percentages values between 30% and 50% [30].

In both extraction techniques, the multivariate second-degree regression analysis indicates high values for R^2^ coefficients (>90%) (Table 3). This means that all of the models have low residual errors when trying to predict the values of either TPC or inhibition % of DPPH, using the independent variables.

### 2.1. Water Bath Extraction

**Figure 1:**
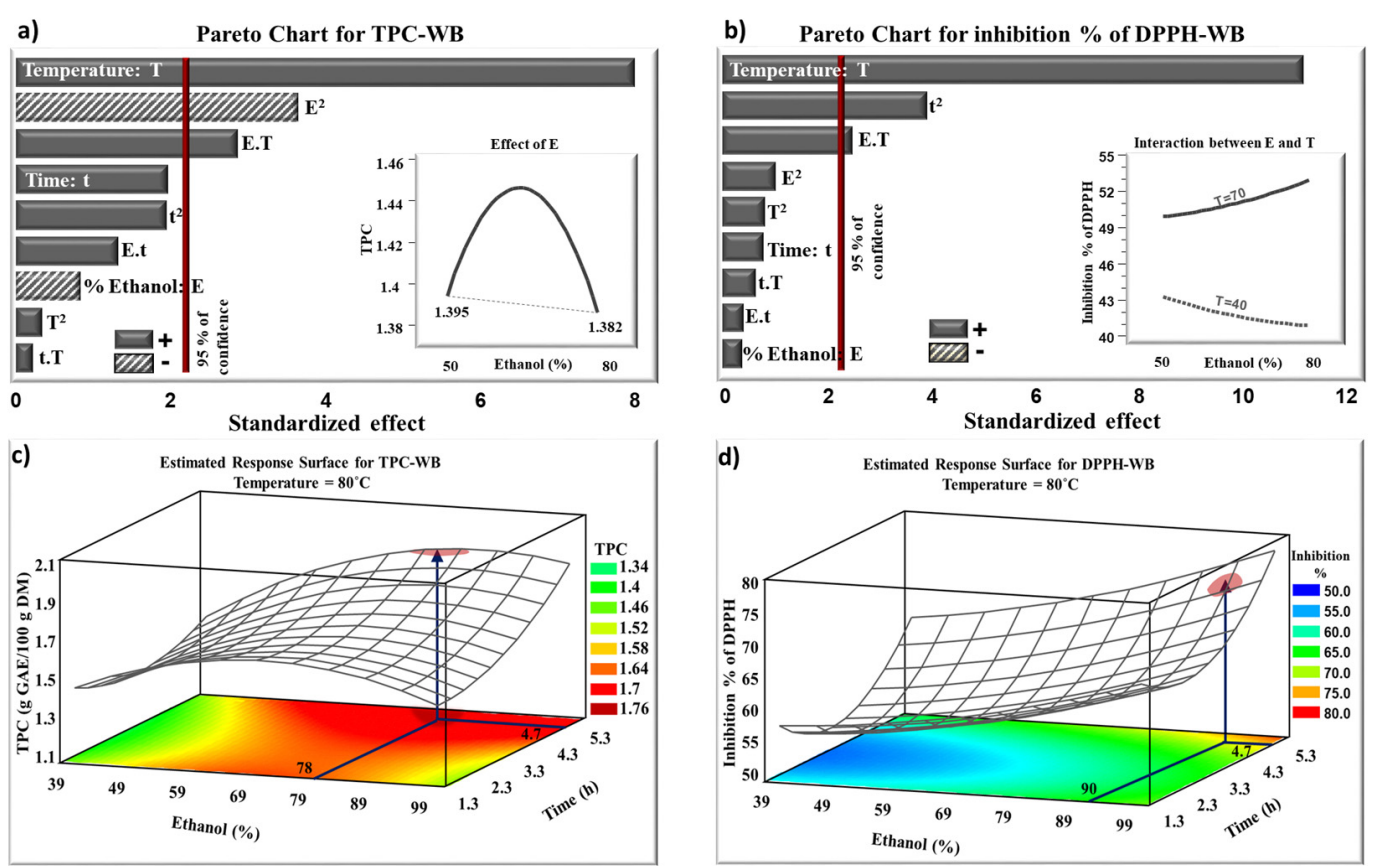
Standardized Pareto charts for TPC (**a**) and the inhibition % of DPPH (**b**). Estimated response surface for TPC (**c**) and the inhibition % of DPPH (**d**). The TPC extracts were obtained after WB extraction.

Pareto charts (Figure 1a) show the positive or negative effects of temperature, time, and ethanol concentration on the extraction of TPC from blood orange peels using WB. The parameters that have the most significant effects (with a confidence level above 95%) correspond to the histograms that cross the vertical line. According to Figure 1a, the temperature had a significant linear positive effect on TPC extraction within the studied domain. The estimated response surface plot, in its three-dimensional illustration, and considering its shape, gives valuable data about the significance of each parameter (Figure 1c). The increase in temperature permitted a higher diffusivity of TPC and improved their transfer into the solvent [31]. Additionally, high temperatures weaken the membrane structure of the cells, which improves the extraction of biomolecules from the peels [14]. TPC are positively affected by the interaction between ethanol and temperature (ET). The increase in ethanol percentage combined with the increase in temperature improved the diffusivity and solubility of TPC in their solvent. Thus, a synergetic effect between ethanol and temperature enhanced the extraction of TPC. However, the TPC content was negatively affected by the quadratic effect of ethanol (E^2^). The increase in ethanol percentage up to a certain value, reduces the dielectric constant of the solvent, which enhances the solubility of TPC and improves their extraction [32]. This is similar to a previous result concerning the effect of ethanol on the extraction of phenolic compounds from flaxseed extracts where the yield of TPC decreased for an ethanol percentage higher than 60% [33]. Higher ethanol concentration may modify the polarity of the solvent and could dehydrate and collapse the plant cells, making difficult the diffusion of biomolecules from the plant matrix to the solvent [34]. In addition, some TPC are soluble in the aqueous phase, whereas others are soluble in organic solvents, which explains the need to use a mixture of ethanol and water to extract higher yields of TPC. The insert in Figure 1a presents the evolution of TPC as a function of ethanol-water percentage and demonstrates that the optimum value of TPC is reached with an intermediate ethanol-water percentage.

Figure 1b,d presents the Pareto chart and the estimated response surface plot for the antiradical activity. The same as for TPC, the DPPH inhibition % was positively affected by the temperature and by the interaction between ethanol percentage and temperature. Studies have shown a correlation between the presence of TPC in extracts and the scavenging activity against the DPPH radical [35,36]. The insert in Figure 1b shows the effect of ethanol percentage on the inhibition % of DPPH at 40 °C (dashed line) and 70 °C (solid line). The observed difference is due to the interaction effect between temperature and ethanol percentage. At 70 °C, a higher amount of TPC is extracted compared to 40 °C, which explains the greater inhibition % of DPPH obtained at this temperature.

### 2.2. Infrared-Assisted Extraction

**Figure 2:**
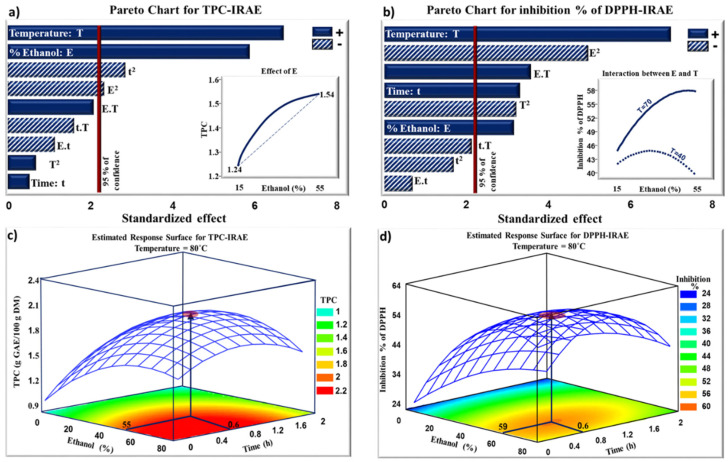
Standardized Pareto charts for TPC (**a**) and the inhibition % of DPPH (**b**). Estimated response surface for TPC (**c**) and the inhibition % of DPPH (**d**). The TPC extracts were obtained after IRAE.

Figure 2a shows the Pareto chart obtained by IRAE. As in WB extraction, TPC solubility increased with temperature, thus facilitating TPC diffusion out of the cells. For the same temperature of 80 °C, the extraction of TPC increased by 20% with IRAE (35% ethanol for 1 h), compared to the conventional WB extraction (65% ethanol for 3 h). This could be associated with cell bursting occurring during IR heating. Ethanol also had positive linear effect on TPC extraction. It acts on the polarity of the medium, which permits to solubilize lipophilic TPC. IR waves are efficiently absorbed by the solvent, which leads to efficient heating and improves the cells rupture for a better extraction of TPC [29,37]. The extraction of TPC with IR is negatively affected by the quadratic effect of time (t^2^). A short period of time was not sufficient for the extraction of TPC. However, longer extraction times could lead to the degradation and oxidation of the extracted TPC, which explains the negative quadratic effect of this parameter [38]. The insert in Figure 2a shows the evolution of TPC as a function of ethanol-water percentage. A maximum value of TPC was obtained when 50% ethanol-water was used as a solvent. Therefore, the use of IR could lead to a reduction in ethanol percentage. Figure 2c presents the evolution of TPC as a function of ethanol percentage and time at a fixed temperature (80 °C). The optimum value of TPC (2.1 g GAE/100 g DM) was obtained after 0.6 h of extraction with 55% ethanol-water. This value was higher than that obtained after 4.7 h of WB extraction (1.78 g GAE/100 g DM) using 78% ethanol-water at the same temperature.

Figure 2b,d shows that temperature, time, and ethanol had linear positive effects on the inhibition percentage of DPPH. The inhibition percentage increased from 34% to 56% when the ethanol percentage, the treatment duration, and the temperature increased from 15%, 0.5 h, and 40 °C to 55%, 1.5 h, and 70 °C, respectively. However, the quadratic effects of ethanol (E^2^) and temperature (T^2^) had negative effects on the inhibition % of DPPH. At high temperatures, the stability of TPC may be negatively affected. Moreover, a possible thermal degradation of TPC already released at low temperatures may have occurred [39]. The insert in Figure 2b presents the evolution of the inhibition % of DPPH as function of ethanol percentage at two different temperatures. The maximum value of 58% inhibition of DPPH was obtained with 50% ethanol-water at 70 °C. A conjugated effect between the increase in ethanol percentage and temperature was observed. The increase in ethanol percentage up to 50% at 70 °C significantly improved the inhibition % of the free radical DPPH. Therefore, the interaction between ethanol and temperature positively affects the antiradical activity of the extracted TPC. A similar behavior was observed with WB (Figure 1b). However, a higher percentage of ethanol-water (80%) is required during WB extraction to obtain only 53% of DPPH inhibition at the same temperature (70 °C).

### 2.3. Multiple Response Optimization: Comparison between the Optimums Obtained with Water Bath Extraction and Infrared-Assisted Extraction

**Figure 3:**
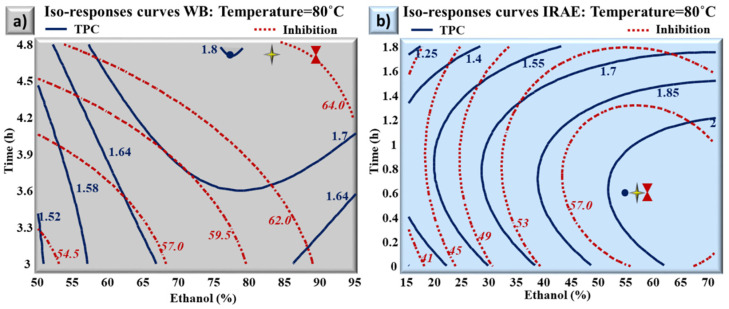
Overlapping for TPC and inhibition percentage of DPPH contour plots in case of WB extraction (**a**) and IRAE (**b**) as obtained at the optimal temperatures for the two responses simultaneously. The blue circle, red hourglass and the four-pointed star represent the optimum of TPC, the optimum of DPPH, and the multiple optimum, respectively.

The optimization of the TPC and the inhibition % of DPPH were conducted separately and the parameters giving the highest quantity of TPC, and the highest inhibition levels of the free radical DPPH were determined. It is important to keep a balance between the concentrations of the TPC and their bioactivity. This is why it is necessary to show the two responses (TPC and inhibition % of DPPH) simultaneously as affected by the combination of the experimental parameters (E, t, T). The simultaneous optimums obtained with the conventional WB extraction (Figure 3a), for both TPC and inhibition % were 1.78 g GAE/100 g DM and 63.5%, respectively. These optimums were found with 83% of ethanol, during 4.7 h at 80 °C. However, IRAE allowed the extraction of 18% more polyphenols (2.1 g GAE/100 g DM) with an inhibition percentage of DPPH of 60%, with 57% of ethanol at 80 °C and for a treatment duration (0.6 h) 7.8 times lower than the WB extraction (Figure 3b).

It is noteworthy to mention that the optimums for TPC and DPPH inhibition converge at the same point and are obtained at close conditions with IRAE, which was not the case with WB. The exactitude of the model was confirmed by repeating the optimal conditions for the optimization of TPC and inhibition percentage for both WB extraction and IRAE (Table 4).

IRAE intensifies the extraction of TPC with shorter time and lower solvent consumption. This result was already shown on the extraction of TPC from grape seeds [37], and pomegranate peels [40]. The efficiency of IRAE compared to conventional extraction technique was also observed in olive leaves where the yield of TPC was improved by more than 30% using IRAE as compared to water bath extraction [41]. In IRAE, the solvent mixture is heated directly, while in conventional WB extraction, a certain period of time is required to heat the container before the heat is transferred to the solution [42]. The efficiency of IRAE can be due to the IR radiation that could stimulate the vibrations in molecules in diverse modes like extending, bending, rocking, and rotating [43]. These vibrations lead to the release of TPC molecules and to an increase in the interactions between the solvent and the active compounds [44], allowing an efficient extraction of TPC from orange peels.

### 2.4. Concentrations and Diversity of Phenolic Compounds Extracted from Orange Peels

HPLC analysis were conducted on the multiple response optimums obtained with WB and IRAE. Table 5 shows the concentration and diversity of the TPC at the optimal points. IRAE selectively extracted caffeic acid and improved the extraction of gallic acid, resveratrol, quercetin and hesperidin by 4.7, 22.6, 17.6, and 24%, respectively, compared to WB extraction. The most remarkable improvement was observed in hesperidin, the principal flavonoid found in orange peels [26], and has an inhibitory effect on food fungal contaminants such as Aspergillus species and mycotoxin production [45]. However, the bioactivity of the extracts could be attributed to the synergetic effects of the extracted TPC. Phenolic quantity depends on the method of extraction and higher TPC yields were obtained with IRAE, which seems to be a promising new technique that intensifies the extraction of bioactive compounds with less time and solvent consumption.

### 2.5. Antifungal Activity

**Figure 4:**
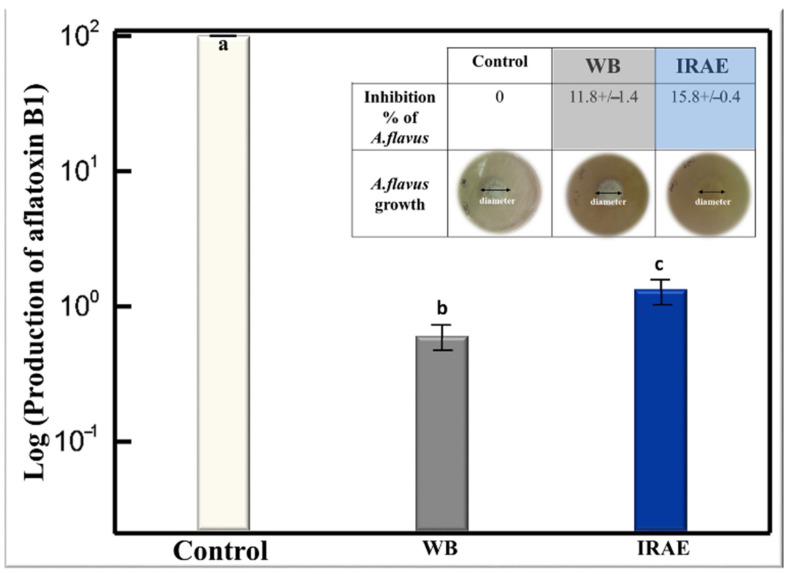
Effect of the simultaneous optimum level of extracts obtained after WB extraction and IRAE on the production of Aflatoxin B1 yield (letters a, b and c indicate significant statistical difference between means). The insert shows the inhibition growth of *A. flavus* by simultaneous optimums extracts obtained after WB extraction and IRAE.

The activities of the extracts obtained at the multiple optimums by WB extraction and IRAE against the growth of *A. flavus* and the production of AFB1 were studied. Figure 4 shows that TPC extracts obtained from WB extraction and IRAE slightly inhibited the growth of *A. flavus* by 11.8% and 15.8%, respectively. However, the inhibition of aflatoxin production was greater than the inhibition of the growth of the fungus. Both TPC extracts obtained from WB extraction and IRAE inhibited AFB1 production by 99.4% and 98.9%, respectively.

AFB1 is synthetized by enzymes encoded within a large cluster of 27 genes. Mycotoxin production is governed by complex environmental signals and different cellular pathways [46]. TPC may inhibit the production of AFB1 by acting at one of the three levels: altering the environmental and physiological signals perceived by the fungi, down regulating the gene expression of the cluster or blocking the activity of certain enzymes involved in the biosynthesis of AFB1 [47].

Extracts from plants or spices, including TPC and essential oils have demonstrated fungicidal and/or anti-toxicogenic properties. In some cases, extracts inhibit the fungal growth and the production of mycotoxin. In other cases, the growth of the fungi is not affected but the production of mycotoxin is partially or totally halted. A variety of flavonoids in tea leaves inhibited the production of AFB1 without affecting the mycelial growth of *A. flavus*. They decreased the production of AFB1 by 99.6%. The inhibition of aflatoxin was attributed to the down-regulations of transcription of genes involved in aflatoxin biosynthesis [48]. Extracts of the plant Garcinia indica inhibited the growth of *A. flavus* and the subsequent production of AFB1 [4]. Eugenol (0.5 mM), the active compound of many essential oils, slightly affected the fungal growth but totally inhibited the production of AFB1. It has been demonstrated that all cluster genes were strongly down regulated in the presence of eugenol. Nineteen out of 27 genes were completely inhibited and the others had 10- to 20-fold reductions in their expression levels [46]. In our case, TPC from blood orange peels are specific inhibitors of AFB1 biosynthesis rather than inhibitors of the fungal growth. This is specifically interesting since the presence of *A. flavus* that cannot produce AFB1 will avoid the contamination of foods with other microbial agents’ producers of other toxins, and will not imbalance the fungal ecology in the field. TPC extracts from orange peels have therefore shown their high potential in the development of anti-aflatoxin agents for food preservation.

## 3. Materials and Methods

### 3.1. Raw Material

Blood orange (*Citrus sinensis*) peels were provided by Balkis Company (Ansariyeh, South Lebanon) and stored at −20 °C until use. Orange peels were manually cut into equal squares of 1 × 1 cm^2^. The moisture content, measured by drying fresh peels at 105 °C to constant weight, was about 76% wet basis.

### 3.2. Extraction Techniques

Intervals of variation in the parameters, notably the percentage of ethanol and the treatment time, were chosen differently for the WB and the IRAE, based on previous studies [30,40,41]. These studies, along with preliminary trials, have proven that the optimal intervals, in terms of TPC yield, for these two techniques are completely distinct. In order to reach the optimal ranges for the WB technique, a higher percentage of ethanol and a longer treatment time than IRAE are required. Within this perspective, the ranges of variation in these two parameters have been established in the following paragraphs.

#### 3.2.1. Water Bath Extraction

Water bath extraction of TPC from blood orange peels was performed with a liquid to solid ratio of 8. Ethanol concentration varied between 40% and 90%, extraction time varied between 1.3 h and 4.7 h, and temperature varied between 30 °C and 80 °C. These values were selected based on preliminary studies, and were optimized by RSM.

#### 3.2.2. Infrared-Assisted Extraction

The infrared-assisted-extraction apparatus, *Ired-Irrad*^®^ (Patent 2017/11-11296L) was used in this purpose. Orange peels were introduced in a round bottom flask with ethanol-water (liquid to solid ratio 8). The flask was placed 1 cm from a ceramic infrared transmitter with a power varying between 70 and 170 W for the irradiation/heating process, linked to a PID control for temperature adjustment. Both the temperature and the voltage can be monitored.

Ethanol concentration varied between 1% and 68%, treatment duration between 0.2 h and 1.8 h and temperature between 29 °C and 80 °C for the IRAE. The optimal conditions for extracting a maximal yield of TPC with the maximum antiradical activity were determined. Extracts were obtained by centrifugation at 6000 rpm for 15 min using a Heraeus Primo R Centrifuge (Thermo Scientific™ Heraeus™, Hanau, Germany). Supernatants were collected and stored at −20 °C until use.

### 3.3. Experimental Designs

Response Surface Methodology (RSM) was used for the optimization of TPC extraction from orange peels by two different techniques. Independent variables were ethanol-water concentration (%), time (t) and temperature (T). The three variables were coded at 5 levels {−α, −1, 0, +1, +α}. The central composite design (2^3^ + star) resulted of twenty experimental points including six repetitions at the central level. Considering three parameters and two responses, experimental data were fitted to obtain a second-degree regression Equation (1) as follows:

(1) $$Y=\beta_{1}+\beta_{2}E+\beta_{3}t+\beta_{4}\text{+}T+\beta_{5}E^{2}+\beta_{6}t^{2}+\beta_{7}{+T}^{2}+\beta_{8}Et+\beta_{9}ET+\beta_{10}tT$$

where Y is the predicted response parameter (TPC or DPPH); β_1_ is the mean value of responses at the central point of the experiment; β_2_, β_3_, and β_4_ are the linear coefficients; β_5_, β_6_, and β_7_ are the quadratic coefficients; β_8_, β_9_, and β_10_ are the interaction coefficients; E is the solvent mixture; t is the treatment time; and T is the treatment temperature. Experimental design and statistical analysis of the results were carried out using STATGRAPHICS*^®^* Centurion XV for windows.

**Figure 5:**
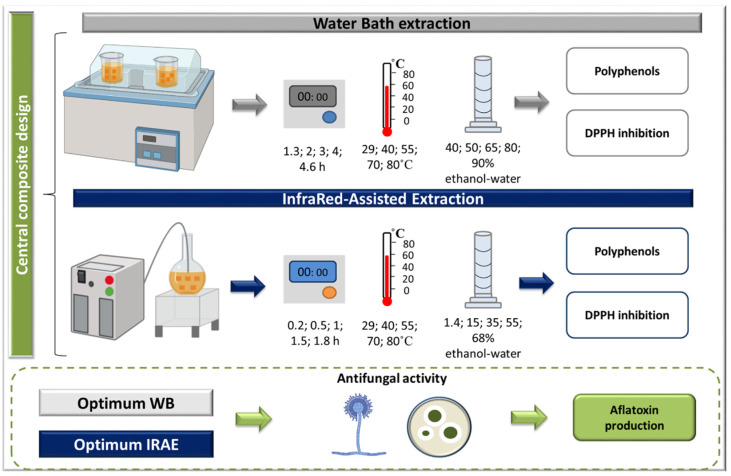
Optimization of TPC extraction from blood orange peels using WB and IRAE. The antifungal activity of the extracts obtained was assessed at optimal conditions.

The experimental protocol is depicted in Figure 5.

### 3.4. Analysis

#### 3.4.1. Total Phenolic Compounds (TPC)

The amount of total TPC extracted from blood orange peels was determined by the Folin-Ciocalteu colorimetrical method [49]. In this method, 1 mL of ten-fold diluted Folin–Ciocalteu reagent (Scharlau, Spain) was added to 0.2 mL of extract. Then, 0.8 mL of sodium carbonate (Na_2_CO_3_) (75 g/L) (BDH, England) were added. The mixture was incubated at 60 °C for 10 min, and then cooled to room temperature. The absorbance was measured at 750 nm by the UV-Vis spectrophotometer (UV-9200, UK). Gallic acid (Sigma-Aldrich, St-Quentin Fallavier, France) was used for the calibration curve. The results were expressed as g of gallic acid equivalent (GAE) per 100 g of dry matter (g GAE/100 g DM).

#### 3.4.2. Antiradical Activity

The free radical scavenging activity was determined by the capacity of the extracted TPC to reduce the free radical, 2,2-diphenyl-1-picrylhydrazyl: DPPH [50]. This method is based on the reduction of the free radical DPPH by phenolic extracts. First, 1.45 mL of DPPH (0.06 mM) (Sigma-Aldrich, St-Quentin Fallavier, France) free radical were added to 50 μL of orange peels extracts or Trolox (control) (Sigma-Aldrich, St-Quentin Fallavier, France). After 30 min of incubation at room temperature, the absorbance was measured at 515 nm. The inhibition percentage of the DPPH free radical was calculated according to Equation (2):

Inhibition Percentage = [(absorbance of control − absorbance of sample)/absorbance of control] × 100(2)

#### 3.4.3. High Performance Liquid Chromatography Analyses

Gallic acid, resveratrol, quercetin, caffeic acid and hesperidin (Sigma-Aldrich, St-Quentin Fallavier, France) were used as standards in high performance liquid chromatography (HPLC) analyses, to identify the TPC in the simultaneous optimums obtained after WB extraction and IRAE. Ultimate 3000 (Dionex, Idtsein, Germany) liquid chromatography apparatus coupled to a diode array detector was used. Before analyses, the samples and standards were filtered through 0.2 µm syringe filters (VWR, Rosny-sous-Bois, France). The chromatography column C18 capillary column 100 × 4.6 mm (Hypersil Gold, Thermo Scientific, MA, USA), was used for all experiments. The temperature of the column was maintained at 40 °C. Water-formic acid solution (95:5) (solvent A) and acetonitrile (solvent B) (HPLC grade, Sigma-Aldrich, St-Quentin Fallavier, France) were used as solvents. A flow rate of 1 mL/min was applied. The HPLC applied method was as follows: 2%–6% of solvent B in 25 min, 6%–15% of solvent B in 15 min, 15%–20% of solvent B in 12 min and 20%–40% of solvent B in 18 min. The injection volume of the sample was 20 μL.

#### 3.4.4. Antifungal Activity

Simultaneous optimums of TPC extracts from orange peels obtained after WB extraction and IRAE were tested against the growth of a toxinogenic fungi *A. flavus* and the toxin secretion of AFB1 from this fungus. *A. flavus* strain NRRL 62477 was used for this purpose.

After the growth of *A. flavus* strain on Czapek yeast extract agar medium (CYA) at 30 °C for 7 days, a spore suspension was prepared. The surface of the prepared culture was scrapped with a sterile Pasteur pipette (Chase Scientific Glass, Inc., Rockwood, TN, USA) and 8 mL of Tween 80 solution (0.005%) were added. A Neubauer hemocytometer (Superior, Marienfeld, Lauda-Konigshofen, Germany) was used to count the spores. The concentration of spores was adjusted to 10^6^ spore/mL and spore suspension was then kept at 4 °C for further use.

A rotavapor was used to evaporate ethanol and concentrate the TPC tested samples. TPC (250 μg) obtained from each WB and IR extract were added to CYA medium. A final volume of 20 mL was poured in petri dishes. For the control culture, 20 mL of CYA were poured in the dish without TPC extracts. Then, 10 µL of the previously prepared spore solution (10^6^ spores/mL) were added in the center of each petri dish. The assays were made in triplicate and all the dishes were left 4 days in the incubator at 28 °C. After this incubation period, the growth inhibition of *A. flavus* was determined. The diameters of the cultures were measured and compared to the negative control. The *A. flavus* inhibition percentage was calculated according to Equation (3):

Inhibition Percentage = [(initial diameter − diameter after incubation)/initial diameter] × 100(3)

#### 3.4.5. AFB1 Extraction and HPLC Analysis

After 7 days of incubation at 28 °C on (CYA) medium, 3 agar plugs (0.5 cm diameter) were removed from different points of the colony for each culture, weighted and placed into 3 mL microtubes. One milliliter of HPLC grade methanol was added to each tube, then the mixture was incubated and shaken for 60 min at room temperature. The mixture was centrifugated for 15 min at 13,000 r.p.m (round per minute). Then, the supernatant phase was recuperated and diluted with 11 mL phosphate buffer. The diluted extract was injected into Aflaprep immunoaffinity columns (R-Biopharm, Glasgow, Scotland) using a syringe for purification. An elution was done by adding 1.5 mL of methanol/acetic acid (98:2, *v/v*) followed by 1.5 mL of HPLC grade water and the total volume was filtered through 0.45 µm filters (Sartorius Stedim Biotech^TM^, PA, USA) then stored at 4 °C before quantification. Aflatoxin B1 quantification was done with a Water Alliance HPLC system using an Utisphere ODB column, C18 (150 × 4.6 mm, 5 µm, 120 Å) (Interchim, Montluçon, France) at 30 °C.

#### 3.4.6. Statistical Analysis

All experiments and measurements were conducted in triplicates. Mean values were calculated and standard deviations were expressed. Significance of the obtained results was evaluated by *p*-value (*p* < 0.05; 95% of confidence level) using analysis of variance (ANOVA) and LSD tests (Least Significant Difference). These statistical analysis and tests were achieved using the software STATGRAPHICS^®^ Centurion XV.

## 4. Conclusions

Our study aimed at valorizing blood orange peels using a simple and low-cost extraction method, *Ired-Irrad^®^*, that has the potential to improve the extraction of TPC. A central composite design optimized the process. Based on the response surface methodology, the optimal TPC extraction parameters were 79 °C for 37 min with an ethanol percentage of 64%. Under these optimized conditions, the experimental yield of TPC and their inhibition percentage matched closely with the predicted results, and TPC yields were 18% higher than those obtained with the conventional solid-liquid extraction. The bioactivity of the extracted TPC was tested on the inhibition of the growth of *A. flavus* and the production of Aflatoxin B1 by the same fungus. Extracted TPC have the potential to be used as natural inhibitors of the carcinogenic Aflatoxin B1 production by *A. flavus*. IR blood orange peels extracts represent an alternative strategy to the use of pesticides to control crop contamination. They can be commercially exploited and applied to food systems. It will be interesting to study the molecular mechanism responsible for the inhibition of Aflatoxin B1 by TPC extracted from blood orange peels.

## 5. Patent

The *Ired-Irrad^®^*, an infrared irradiation apparatus, was designed and patented in collaboration between Faculty of Sciences at USJ and Faculty of Arts and Sciences at University of Balamand. Lebanese patent 2017/11-11296L, granted on 29 November 2017.

## Figures and Tables

**Table 1:** RSM central composite design for three parameters (real and coded values) of five levels and the experimental responses (TPC and inhibition % of DPPH) of **WB** extraction from blood orange peels.

	Run	Ethanol (%) Real [Coded] Value	Time (h) Real [Coded] Value	Temperature (°C) Real [Coded] Value	TPC (g GAE/100 g DM)	Inhibition % of DPPH
**Factorial Design**	1	50 [−1]	2 [−1]	40 [−1]	1.36	46.04
	2	80 [+1]	2 [−1]	40 [−1]	1.20	42.45
	3	50 [−1]	4 [+1]	40 [−1]	1.37	44.24
	4	80 [+1]	4 [+1]	40 [−1]	1.33	43.50
	5	50 [−1]	2 [−1]	70 [+1]	1.48	50.52
	6	80 [+1]	2 [−1]	70 [+1]	1.55	55.01
	7	5 0 [−1]	4 [+1]	70 [+1]	1.54	52.17
	8	80 [+1]	4 [+1]	70 [+1]	1.67	55.75
**Center Points**	9	65 [0]	3 [0]	55 [0]	1.40	45.59
	10	65 [0]	3 [0]	55 [0]	1.38	44.69
	11	65 [0]	3 [0]	55 [0]	1.50	46.49
	12	65 [0]	3 [0]	55 [0]	1.46	46.93
	13	65 [0]	3 [0]	55 [0]	1.44	45.29
	14	65 [0]	3 [0]	55 [0]	1.47	49.77
**Star Points**	15	39.8 [−α]	3 [0]	55 [0]	1.33	48.88
	16	90.2 [+α]	3 [0]	55 [0]	1.24	48.06
	17	65 [0]	1.3 [−α]	55 [0]	1.48	51.27
	18	65 [0]	4.7 [+α]	55 [0]	1.50	53.29
	19	65 [0]	3 [0]	29.8 [−α]	1.28	37.64
	20	65 [0]	3 [0]	80.2 [+α]	1.58	58.74

**Table 2:** RSM central composite design of three parameters (real and coded values) of five levels and the experimental responses (TPC and inhibition % of DPPH) of **IRAE** from blood orange peels.

	Run	Ethanol (%) Real [Coded] Value	Time (h) Real [Coded] Value	Temperature (°C) Real [Coded] Value	TPC (g GAE/100 g DM)	Inhibition % of DPPH
**Factorial Design**	1	15 [−1]	0.5 [−1]	40 [−1]	0.95	34.75
	2	55 [+1]	0.5 [−1]	40 [−1]	1.22	36.06
	3	15 [−1]	1.5 [+1]	40 [−1]	1.12	47.54
	4	55 [+1]	1.5 [+1]	40 [−1]	1.30	42.95
	5	15 [−1]	0.5 [−1]	70 [+1]	1.18	42.79
	6	55 [+1]	0.5 [−1]	70 [+1]	1.79	54.10
	7	15 [−1]	1.5 [+1]	70 [+1]	1.19	44.26
	8	55 [+1]	1.5 [+1]	70 [+1]	1.60	56.39
**Center Points**	9	35 [0]	1 [0]	55 [0]	1.39	54.10
	10	35 [0]	1 [0]	55 [0]	1.47	55.90
	11	35 [0]	1 [0]	55 [0]	1.34	48.85
	12	35 [0]	1 [0]	55 [0]	1.50	52.95
	13	35 [0]	1 [0]	55 [0]	1.48	47.38
	14	35 [0]	1 [0]	55 [0]	1.48	52.62
**Star Points**	15	1.36 [−α]	1 [0]	55 [0]	1.17	39.34
	16	68.6 [+α]	1 [0]	55 [0]	1.52	45.74
	17	35 [0]	0.16 [−α]	55 [0]	1.27	46.39
	18	35 [0]	1.84 [+α]	55 [0]	1.34	51.64
	19	35 [0]	1 [0]	29.8 [−α]	1.21	36.39
	20	35 [0]	1 [0]	80.2 [+α]	1.90	55.57

**Table 3:** The second order regression equation for TPC and inhibition % of DPPH of each extraction technique and the R-squared of each equation.

Extraction Technique	R^2^ (Percent)	Equation
**WB**	90.45	TPC = 1.55 + 0.01E − 0.24t −0.01T − 0.0002E^2^ + 0.002Et + 0.0002ET + 0.02t^2^ + 0.0002tT + 0.00002T^2^
	93.26	Inhibition % of DPPH = 87.46 − 0.69E − 13.03t − 0.35T + 0.002E^2^ + 0.02Et + 0.007ET + 1.81t^2^ + 0.03tT + 0.002T^2^
**IRAE**	91.08	TPC = 0.22 + 0.01E + 1.12t + 0.002T − 0.0001E^2^ − 0.004Et + 0.0002ET − 0.28t^2^ − 0.007tT + 0.00007T^2^
	92.24	Inhibition % of DPPH = −15.95 + 0.17E + 30.92t + 1.31T − 0.009E^2^ − 0.06Et + 0.01ET − 4.69t^2^ − 0.27tT − 0.01T^2^

**Table 4:** Predicted and experimental results for TPC and inhibition %, at the multiple optimum conditions for WB extraction and IRAE.

Extraction Technique (Optimal Conditions)	Multiple Optimum	Predicted Values	Experimental Values
**WB** **(T = 80 °C t = 4.7 h E = 83%)**	TPC g GAE/100 g DM	1.78	1.77 ± 0.040
	Inhibition %	63.5	61.7 ± 0.32
**IRAE** **(T = 80 °C t = 0.6 h E = 57%)**	TPC g GAE/100 g DM	2.1	2.2 ± 0.043
	Inhibition %	60	61.5 ± 0.24

**Table 5:** TPC (mg/100 g DM) obtained in the simultaneous optimums after WB extraction and IRAE.

Phenolic Compound (mg/100 g DM)	WB	IRAE
**Gallic acid**	2.33 ± 0.20	2.44 ± 0.042
**Resveratrol**	28.46 ± 2.16	34.89 ± 0.54
**Quercetin**	5.50 ± 0.79	6.47 ± 0.58
**Caffeic acid**	nd	0.16 ± 0.050
**Hesperidin**	286.67 ± 0.60	355.60 ± 0.89

## Data Availability

Not applicable.

## Sample Availability

Not available.
